# Curcumin prophylaxis mitigates the incidence of hypobaric hypoxia-induced altered ion channels expression and impaired tight junction proteins integrity in rat brain

**DOI:** 10.1186/s12974-015-0326-4

**Published:** 2015-06-06

**Authors:** SKS Sarada, M Titto, P Himadri, S Saumya, V Vijayalakshmi

**Affiliations:** Haematology Division, Defence Institute of Physiology and Allied Sciences, Lucknow Road, Timarpur, Delhi 110054 India

**Keywords:** Curcumin, Hypoxia, Cerebral edema, NF-κB, HIF-1α, Fluid reabsorption, Na^+^/K^+^-ATPase, ENaC, Tight junction proteins, Inflammation

## Abstract

**Background:**

The present study was proposed to elucidate the prophylactic role of curcumin in the prevention of hypoxia-induced cerebral edema (HACE).

**Methods:**

Rats were exposed to simulated hypobaric hypoxia at 7620 m for 24 h at 25 ± 1 °C. Transvascular leakage, expression of transcriptional factors (nuclear factor-kappa B (NF-κB) and hypoxia inducible factor 1 alpha (Hif-1α) and also the genes regulated by these transcriptional factors, sodium potassium-adenosine triphosphatase (Na^+^/K^+^-ATPase) and endothelial sodium channel (ENaC) levels and brain tight junction (TJ) proteins like ZO-1, junctional adhesion molecule C (JAMC), claudin 4 and claudin 5 levels were determined in the brain of rats under hypoxia by Western blotting, electro mobility shift assay, ELISA, immunohistochemistry, and histopathology along with haematological parameters. Simultaneously, to rule out the fact that inflammation causes impaired Na^+^/K^+^-ATPase and ENaC functions and disturbing the TJ integrity leading to cerebral edema, the rats were pre-treated with curcumin (100 mg/kg body weight) 1 h prior to 24-h hypoxia.

**Results:**

Curcumin administration to rats, under hypoxia showed a significant decrease (*p* < 0.001) in brain water content (3.53 ± 0.58 wet-to-dry-weight (W/D) ratio) and transvascular leakage (136.2 ± 13.24 relative fluorescence units per gram (r.f.u./g)) in the brain of rats compared to control (24-h hypoxia) (7.1 ± 1.0 W/D ratio and 262.42 ± 24.67 r.f.u./g, respectively). Curcumin prophylaxis significantly attenuated the upregulation of NF-κB (*p* < 0.001), thereby leading to concomitant downregulation of pro-inflammatory cytokine levels (↓IL-1, IL-2, IL-18 and TNF-α), cell adhesion molecules (↓P-selectin and E-selectin) and increased anti-inflammatory cytokine (↑IL-10). Curcumin stabilized the brain HIF-1α levels followed by maintaining VEGF levels along with upregulated Na^+^/K^+^-ATPase and ENaC levels (*p* < 0.001) under hypoxia. Curcumin restored the brain ZO-1, JAMC, claudin 4 and claudin 5 levels (*p* < 0.001) under hypoxia. Histopathological observations revealed the absence of edema and inflammation in the brain of rats supplemented with curcumin.

**Conclusions:**

These results indicate that curcumin is a potent drug in amelioration of HACE as it effectively attenuated inflammation as well as fluid influx by maintaining the tight junction proteins integrity with increased ion channels expression in the brain of rats under hypoxia.

## Background

High-altitude cerebral edema (HACE) occurs in un-acclimatised individuals who ascend rapidly from low to high altitudes. HACE is the result of swelling of the brain tissue from fluid leakage and almost always begins as acute mountain sickness. The symptoms include headache, nausea, insomnia, ataxia, loss of memory, hallucinations and coma. The exact mechanism of the pathogenesis of HACE is not yet fully understood. HACE is uncommon but sometimes fatal and constitute about 0.5–1 % at 4000–5000 m [1, 2] and 3.4 % in those who suffer from acute mountain sickness (AMS). The lowest altitude at which a case of HACE was reported was 2100 m [3]. Less commonly, it also occurs at extreme altitude (over 7000 m) in climbers apparently well acclimatised [4]. Basnyat et al. [5] by using a questionnaire reported that Vedic pilgrims in Nepal experienced an astonishing figure of 31 % of HACE at 4300 m. Several authors have postulated different mechanisms to explore the pathophysiology of HACE [1, 6, 7] viz., (i) mechanical factors increase intracellular pressure hence cause vasogenic edema, (ii) hypoxic ventilator response resulting in hypocapnea which causes vasoconstriction, and (iii) cytotoxic edema due to failure in sodium potassium-adenosine triphosphatase (Na^+^/K^+^-ATPase). Along with these, many chemical mediators have been implicated in developing HACE, such as (i) free-radical formation could directly damage vessel basement membranes causing vasogenic edema; (ii) accumulation of hypoxia-inducible factor 1 alpha (HIF-1α) and subsequent upregulation of vascular endothelial growth factor (VEGF) could contribute further basement membrane damage [8]; (iii) local hyperkalaemia could trigger calcium-mediated nitric oxide release, which in turn act on vascular smooth muscle to cause vasodilatation; and (iv) neuronal-mediated adenosine release leading to further enhanced vasodilatation, this perhaps activates the trigeminovascular system causing headache. All these changes lead to micro haemorrhage causing more fluid accumulation in the brain.

The epithelial sodium channel endothelial sodium channel (ENaC) and Na^+^/K^+^-ATPase plays an essential role in the regulation of transepithelial sodium and fluid balance in various tissues including the brain. However, there are no published studies to our knowledge that have studied changes in the ENaC and Na^+^/K^+^-ATPase activity by prophylactic administration of curcumin. The cerebral micro vessel endothelial cells that form the blood brain barrier (BBB) have tight junctions (TJ) that are critical for maintaining brain homeostasis and low permeability. TJ are complexes of plasma membrane proteins that connect to the cytoskeleton architecture via membrane-associated accessory proteins [9]. Claudins and occludin are integral transmembrane proteins which interact with plasma membranes of adjacent cells forming the TJ barrier [10, 11]. Similarly, Zonula occludin proteins (ZO-1 and ZO-2) are membrane-associated proteins among tight junction complexes that are anchored to the cytoskeleton architecture via occludin [12, 13]. Although TJ complexes have been well established, little is known about alternations in those proteins under pathological insult, i.e. hypoxia and how curcumin affects this architecture under hypoxia has not been reported. However, it is well known that hypoxia induce an inflammatory response [14] that could further impair organ function through leukocytes accumulation and increased capillary leakage. In our recent study, we have reported that oxidative stress and nuclear factor-kappa B (NF-κB) contribute in causing edema in the brain under hypoxia [15]. The best treatment for HACE is immediate descent. A portable hyperbaric chamber or oxygen administration may also give relief. Pharmacological treatment of HACE is with dexamethasone which blocks VEGF, therefore reversing the hypoxia-induced brain edema [6]. As a chemoprophylaxis for HACE, acetazolamide and dexamethasone are recommended. Acetazolamide causes both metabolic acidosis and increased ventilation followed by reduction in cerebrospinal fluid [16]. Dexamethasone, a glucocorticoid, reduces the release of cytokines and capillary permeability in the brain [17]. It seems very clear that the drugs that are recommended for HACE are able to reduce the oxidative stress and inflammation. Therefore, identification of a potent prophylactic agent which can act both as an antioxidant and anti-inflammatory molecule might also reduce high-altitude cerebral edema. Plant-based formulations like *Ginko biloba* extract has been used to treat AMS and HACE [2]. Its mechanism of action is unclear, but it is believed to work by scavenging free radicals. Traditional plant-derived medicines are safe, less expensive and also available all the time. Best illustrated of such a molecule is curcumin, a derivative of turmeric used for centuries to treat a wide variety of inflammatory conditions.

Curcumin is a diferulomethane derived from the Indian spice plant (*Curcuma longa Linn*) turmeric (popularly called “curry powder”) that has been shown to interfere with cell signalling pathways, apoptosis, proliferation, angiogenesis, metastasis and inflammation [18]. The uses of turmeric, for treatment of different inflammatory diseases, have been described in Ayurveda and in traditional Chinese medicine since thousands of years. The active component of turmeric responsible for this activity is curcumin, which was identified almost two centuries ago. Modern science has revealed that curcumin mediates its effects by modulating several important molecular targets. Curcumin has been shown to upregulate glutathione transferase [19], inhibit cytochrome P450 enzymes [20] and reduce oxidative stress [21] and inflammation [22]. Protein binding by curcumin has been reported to induce the degradation of p50 in the nuclear factor-kB complex [23] thus downregulating NF-κB production. Therefore, taking into consideration about the earlier reports, in the present study, we tried to explore the molecular aspects of association between hypoxia and inflammation, i.e. HIF-1α vs NF-κB in hypoxia-induced cerebral edema. How these two transcriptional factors have been positively correlated with altering the ion channels expression leading to failure in fluid reabsorption and loss in tight junction proteins integrity causing increased vascular leakage in the brain of rats under hypoxia? Moreover, in the present study, we tried to ameliorate these effects by the potent phytochemical molecule curcumin.

## Materials and methods

### Animals

The experiments were conducted using male rats (Sprague Dawley strain) with an average body weight of 150–200 g. The reason for selecting male Sprague Dawley rats was that males are found to have a significantly higher incidence of acute mountain sickness than females [24]. All rats were obtained and maintained in the Institute’s animal house, and they were exposed to 12:12-h light-to-dark cycles each at 25 °C. The rats were provided with food and water *ad libitum*. All animal procedures and experimental protocols were approved by institutional Animal Ethics Committee (Authorisation No: 27/1999/CPCSEA) and followed the standards set forth in the guide for the care and use of laboratory animals (National Academy of Science, Washington, DC, USA).

### Experimental setup

The experiment was carried out in two phases:

Phase I. Dose-dependent studies of curcumin on brain transvascular leakage were carried out in rats. The rats were divided into six groups each containing six rats. Group 1 served as control (normoxia or 0 h) received only vehicle; group 2 exposed to hypoxia for 24-h received only vehicle; group 3 animals were supplemented with curcumin at 50 mg/kg body weight (BW), 1 h prior to hypoxia exposure; group 4 animals were supplemented with curcumin at 100 mg/kg BW, 1 h prior to hypoxia exposure; group 5 animals were supplemented with curcumin at 200 mg/kg BW, 1 h prior to hypoxia exposure; and group 6 animals were supplemented with curcumin at 300 mg/kg BW, 1 h prior to hypoxia exposure.

**Fig. 1:**
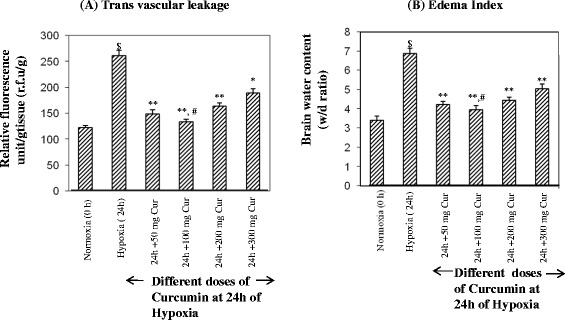
Optimization of curcumin dose in hypoxia-exposed rats. **a** Transvascular leakage and (**b**) the brain water content. The rats were pre-treated with curcumin at 50, 100, 200 and 300 mg/kg BW orally 1 h prior to hypoxia exposure at 7620 m for 24 h. The optimum dose of curcumin was found to be 100 mg/kg BW where there was minimum transvascular leakage, and the brain water content and was found to be significantly (*p* < 0.05) different from other doses of curcumin. Values are mean ± SD (*n* = 6). ^$^ *p* < 0.001 compared to normoxia; **p < 0.001 compared to 24-h hypoxia; *p < 0.01 compared to 24-h hypoxia; ^#^ *p* < 0.05 compared to other groups. *N* normoxia, *H* hypoxia, *Cur* curcumin

Phase II. The dose-dependent studies of curcumin from phase I studies revealed that 100 mg/kg BW showed significantly reduced edema index compared to other doses tested and hence, considered as optimum dose (Fig. 1a, b). Therefore, the rest of the experiments were carried out using 100 mg cur/kg BW in 24 rats. In the second phase of the experiment, a total of 24 rats were used. The rats were divided into four groups containing six rats each. Group I served as control (normoxia or 0 h) receiving only vehicle, group II (hypoxia) received only vehicle and exposed to hypoxia for 24 h. Curcumin administration to rats was carried out 1 h prior to hypoxic exposure. Group III (normoxia + curcumin) was administered with curcumin at 100 mg/kg BW; group IV (hypoxia + curcumin) was administered with curcumin at 100 mg/kg BW, and then exposed to hypoxia for 24 h.

### Optimization of curcumin dose

Extensive scientific research on curcumin demonstrated its anti-inflammatory action. Curcumin isolated from the alcoholic extract of turmeric has been shown to be a useful anti-inflammatory agent. Curcumin was reported to stabilise lysosomal membrane and cause uncoupling of oxidative phosphorylation besides having strong oxygen radical scavenging activity, which was responsible for its anti-inflammatory property [25]. In various animal studies, a dose range of 100–200 mg/kg BW exhibited good anti-inflammatory activity and found to have negligible adverse effect on human systems [25]. In sub-acute toxicity experiments, no significant toxic side effects were observed in rats when the extract was administered for 4 weeks at a dose level of 1–2 g/kg [25] and even for 90 days at a dose level of 1.8 g/kg per day [26]. Recent reports had also demonstrated an anti-inflammatory activity of curcumin in acute and chronic models of inflammation in rats and mice [21, 25]. The average intake of turmeric in the diet in India is approximately 2–2.5 g in a 60-kg individual which corresponds to an intake of approximately 60–100 mg of curcumin daily [27]. Curcumin is lipophilic in nature and it is transported in the blood by binding tightly to serum albumin [28] and several other molecules in the body. Regarding bioavailability of curcumin Ravindranath and Chendrashekara [29] showed that about 90 % of curcumin was found in the stomach and small intestine in 30 min up on oral administration of 400 mg of curcumin. However, very small quantity in the liver and kidney (<20 μg/tissue) was absorbed from 15 min to 24 h after administration. These authors further reported that 60–66 % of curcumin absorbed remained constant regardless of the dose, indicating that administration of higher concentration of curcumin to rats does not result in higher absorption. Research reveal that the poor bioavailability of curcumin can be overcome by combination of curcumin with piperine (a component from black pepper), liposomal or phospholipid complexes or development of nanocurcumin or curcumin analogues, etc. [30] which bring significant delay in its elimination. Although dexamethasone at 8 mg/day, in divided doses, can prevent AMS and HACE, but the potential side effects have restricted its use to those for whom acetazolamide is contraindicated and to rescue workers on whom rapid ascent is mandatory and unavoidable. Steroid prophylaxis has benefits without altering physiological variables such as peripheral oxygen saturation [31]. It is normally used to treat, rather than to prevent, AMS and HACE. Pharmacological interventions with respect to prevention and treatment for HACE are limited to curcumin as a prophylactic drug of choice. Therefore, considering our phase I experimental results, as well as the previous reports on its dose response, we had considered 100 mg/kg BW as the optimum dose for further experimentation. We did not find any adverse effects in any one of the experimental rats among the different doses of curcumin tested under hypoxia

### Administration of curcumin

The drug curcumin was purchased from Sigma Aldrich (St. Louis, MO, USA). The drug was freshly dissolved in DMSO (0.5 %) and administered orally to rats, 1 h prior to hypoxia exposure at 7620 m for 24-h duration.

### Details of hypoxia exposure

Dose-dependent studies were carried out in our earlier studies [15], wherein the maximum increase in cerebral transvascular leakage was obtained during 24 h of hypoxia exposure. Therefore, in the present study, the rats were exposed to hypoxia for 24-h duration and thereby the prophylactic effect of curcumin against high-altitude cerebral edema was studied.

The rats were exposed to simulated altitude of 7620 m (280 mm Hg) at 25 ± 1 °C in a hypobaric chamber. Fresh air was flushed continuously into the chamber at a rate of 4 l/h, and humidity of the chamber was maintained at 60 ± 2 %. The partial pressure of arterial oxygen in control rats was found to be 95 ± 2 mm Hg, and in hypoxia exposed rats, it was found to be 38 ± 2 mm Hg, indicating that the rats were exposed to reduced levels of partial pressure of oxygen in the hypobaric chamber. The animals were supplied with adequate quantities of food and water during exposure to hypobaric hypoxia.

### Determination of the brain water content and vascular permeability

#### Brain water content

To quantify the brain water content in normoxia (control) and hypoxia-exposed animals, the wet weight of the brain tissue was taken immediately after removal. The samples were dried at 110 °C for 24 h and reweighed to give the dry weight. The brain water content was calculated by taking the wet-to-dry-weight ratio and expressed as W/D ratio [6].

#### Vascular permeability

To quantify the vascular permeability in the brain of rats, 200 μl of sodium fluorescein dye (Sigma-Aldrich, St. Louis, MO, USA) at a concentration of 5 mg/kg BW in phosphate buffer saline (PBS) was injected through the tail vein in both control and hypoxia-exposed rats. In brief, 30 min before the predetermined time of hypoxia exposure, the rats were injected with the sodium fluorescein dye. Later, the animals were placed back in the hypoxic chamber up to the determined time. After exposure, the animals were anesthetized and then 30 min later, refrigerated PBS was infused through left ventricle to remove the fluorescent tracer from the vascular bed. Subsequently, the whole brain was removed, washed with cold saline (0.9 % NaCl) and divided into two equal parts. One part was kept in 3 % formamide (Sigma-Aldrich, St. Louis, MO, USA) and left undisturbed for about 18 h at room temperature. The other part was kept in an oven at 55–60 °C for 48 h. After 18 h, the tissues in formamide were centrifuged for 10 min at 3000 rpm. Then, the fluorescence of the tissue supernatant was collected in a dark room, and a reading was taken spectrofluorometrically at 485 nm excitation and 530 nm emission and results were presented as relative fluorescence unit (r.f.u.)/g of tissue [32].

### Haematological parameters

Blood was collected in ethylene diamine tetra acetic acid (EDTA) from the retro orbital plexus after 24 h hypoxia in all the six groups and were used for various haematological analyses, viz white blood corpuscles (WBC), red blood corpuscles (RBC), lymphocytes, granulocytes, monocytes, mean cell volume (MCV) and haemoglobin (Hb). The analysis was carried out using a Sysmex Model F820 hematology cell analyzer (Toa Medical Electronics Co. Ltd., Kobe, Japan).

### Estimation of redox-sensitive transcription factor, NF-κB, activation studies

#### Sample preparation

The whole brain was removed and rinsed in cold saline and made into 10 % ice-cold homogenate (in 0.154 M KCl) for estimating various biochemical parameters. Nuclear cytoplasmic protein extracts were prepared using a modified method as described earlier [33]. Briefly, the brain tissues were homogenised in ice-cold buffer containing 10 mM HEPES, pH 7.9, 10 mM KCl, 0.1 mM EDTA, 1.5 mM MgCl_2_, 1 mM dithiothreitol, 0.5 mM phenlymethysulfonyl fluoride, 10 μl/ml of cocktail (protease inhibitors antipain, chymostatin, pepstatin and leupeptin). Cytoplasmic fraction was centrifuged at 8000 rpm (Sigma 3/18 K-Germany) for 4 min at 4 °C, and the nuclear fraction was reconstituted in a buffer containing 20 mM HEPES, pH 7.9, 400 mM NaCl, 1 mM phenylmethylsulfonyl fluoride, and 10 μl/ml of cocktail. The nuclear suspension was centrifuged at 14,000 rpm for 5 min, and the supernatant containing the nuclear extracts were collected and stored at −80 °C for further transcription factor expression studies.

### Electrophoretic mobility shift assay (EMSA) for NF-κB and HIF-1α DNA-binding activity

Nuclear extracts prepared from rat brain tissues were used in this study. The EMSA for NF-κB was carried out using a commercial kit (Pierce, Winooski, VT, USA). The binding mixture (25 ml) containing 10 μg protein of nuclear extract and 1 μg of poly (dI-dC) was incubated in a Tris-EDTA buffer (10 mM Tris–HCl, pH 7.4, 50 mM NaCl, 50 mM KCl, 1 mM MgCl_2_, 1 mM EDTA, 5 mM DTT) placed on ice for 15 min. Later, 10 ηg of biotinylated double-stranded consensus oligonucleotide for EMSA of NF-κB binding (5′-AGT TGA GGG GAC TTT CCC AGG C-3′); a mutant DNA sequence (5′-GCC TGG GAA AGT CCC CTC AAC T-3′) and for EMSA of HIF-1α binding (5′-TCTGT**ACGTG**ACCACACTCACCTC-3′); a mutant DNA sequence (5′-TCTGT**AAAAG**ACCACACTCACCTC-3′) was added and incubated at room temperature (RT) for 30 min. Then, DNA-protein complexes were separated on native 6 % polyacrylamide DNA retardation gel and electro blotted onto positively charged nylon membranes. Biotinylated DNA/protein complexes were detected with peroxidase conjugated streptavidin and a chemiluminescent substrate kit (Pierce, Winooski, VT, USA).

### Western blotting studies

From both nuclear and cytoplasmic brain extracts, 50 μg of protein was separated on 10 % sodium dodecyl sulfate polyacrylamide gel electrophoresis (Bio-Rad Laboratories, Inc., Hercules, CA, USA) and electro blotted onto nitrocellulose membranes (Merck Millipore, Billerica, MA, USA). Membranes were then blocked in 0.1 % phosphate buffered saline, 0.1 % Tween 20 (PBST; pH 7.4) with 5 % non fat milk for 2 h at room temperature and thoroughly washed with PBST. The membranes were further incubated with primary antibodies (NF-κB p65; IL-1 α, IL-18, TNF-α, P-selectin, E-selectin, ENaC, α_1_Na^+^/K^+^-ATPase, HIF-1α, VEGF, JAMC and ZO-1; Santa Cruz Biotechnology, Santa Cruz, CA, USA) diluted in PBST for 2 h at room temperature. The membranes were washed with PBST and then probed with anti-mouse-IgG-HRP conjugate (1:50,000) for 1 h. Finally, the membranes were washed again with PBST and developed using a kit (Chemiluminescence substrate; Sigma-Aldrich, St. Louis, MO, USA) and bands were visualized on X-ray film (Kodak, Rochester, NY, USA). Densitometric analysis was carried out by using the Gel Doc system (UVP, Bio Imaging system, Cambridge, UK). To further ensure that equal concentrations of protein had been loaded, α-tubulin and β-actin protein expressions were determined from the brain homogenate by Western blotting.

### Cytokine analysis: enzyme-linked immunosorbent assay (ELISA)

ELISA was used to measure IL-2 (Ray Biotech, Norcross, GA, USA) and IL-10 (BD Bioscience, San Jose, CA, USA) protein levels from plasma obtained from both normoxia- and hypoxia-exposed animals. The assay was performed according to the protocols given by the manufacturer. Sample observations were read with an ELISA plate reader (BMG Lab Tech, Ortenberg, Germany) adjusted to 450 nm and the concentrations were determined based on rat IL-2 and IL-10 standards provided by the manufacturers.

### Immunohistochemistry

Immunohistochemistry was performed in normoxia- or hypoxia-exposed rat brain tissue as described by Beytut et al. [34]. Thin sections of the brain were taken and fixed with paraformaldehyde. Endogenous peroxidase activity was blocked with hydrogen peroxide (3 %) in distilled water for 30 min. The sections were incubated with PBS, (pH 7.2) for 5 min and subsequently placed into 0.05 % Trypsin EDTA for 20 min for antigen retrieval. After washing with PBS, the sections were incubated with 5 % normal goat serum for 60 min at RT. The sections were then incubated with each of the primary antibodies (claudin 4 and claudin 5 from Santa Cruz Biotechnology, Santa Cruz, CA, USA) for overnight at 4 °C. Following 4–5 times washing with PBST, the sections were incubated with HRP conjugated goat anti-rabbit IgG in PBST for 60 min at RT. Secondary antibodies were supplied by Sigma-Aldrich, St. Louis, MO, USA. Labelling was visualized with 3,3′-diaminobenzidine (DAB) as the chromogen. The images were captured by using Olympus BX51TF (Olympus Corporation, Centre Valley, PA, USA).

### Histopathological processing and evaluation of the brain samples

After the stipulated time period of 24 h of hypoxia exposure at an altitude of 7620 m, rats were anesthetized with ketamine/xylazine (70 and 6 mg/kg, i.p., respectively) and transcardially perfused with phosphate buffered saline (PBS; pH 7.4 at room temperature) followed by fixation in ice-cold 4 % paraformaldehyde overnight. Later, whole brains were extracted, immersed in 4 % paraformaldehyde for 24 h at room temperature followed by dehydration through graded alcohol series and embedded in paraffin. After dehydration in alcohol and clearing in xylene, approximately 40-μm-thick coronal sections from cerebral cortex were made and the selected sections were stained with haematoxylin and eosin for detection of brain edema and inflammatory cells like polymorphonucleocytes, monocytes and macrophage-like cells. The photomicrographs of region of interests were captured with a digital camera (Nikon, Tokyo, Japan) attached to a light microscope (Olympus Corporation, Centre Valley, PA, USA).

### Statistical data analysis

Results were expressed as mean ± SD. Comparisons between normoxia and 24-h hypoxia-exposed animals were assessed by using a *t* test applying the Bonferroni correction, and differences between hypoxia and curcumin administered hypoxia-exposed groups of animals were assessed by using one-way ANOVA with the Student-Newman-Keuls test for multiple comparisons among groups. Differences were considered statistically significant for *p* < 0.05. All statistical tests were performed with the SPSS statistical software, version 12.0 for Windows (SPSS Inc., Chicago, IL, USA).

## Results

### Determination of cerebral edema

#### Determination of vascular permeability

Dose-dependent studies of curcumin were carried out to find out the optimum dose at which minimum vascular permeability would be obtained. We have quantified the vascular leakage of the brain in rats supplemented with different doses of curcumin, i.e. 50, 100, 200 mg and 300 mg cur/kg BW. Figure 1a represents the relative fluorescence unit/g (r.f.u./g) in the brain of rats supplemented with different doses of curcumin under hypoxia. Curcumin at 50 mg cur/kg BW showed significant decrease (*p* < 0.001) in vascular leakage in the brain of rats exposed to hypoxia compared to hypoxia exposed rats without curcumin. Whereas rats exposed to hypoxia supplemented with 100 mg cur/kg BW showed further significant decrease (*p* < 0.001) in transvascular leakage (136.2 ± 13.24 r.f.u./g) compared to control (262.42 ± 24.67 r.f.u./g; hypoxia 24 h). Further increase in curcumin dose, i.e. 200 and 300 mg cur/kg BW, did not show further improvement in reducing the vascular permeability in the brain of rats exposed to hypoxia, compared to 50 mg curcumin administered hypoxia-exposed rats (Fig. 1a)

#### Determination of the brain water content

The brain water content was determined by calculating the wet-weight-to-dry-weight ratio of the brain tissue of rats, supplemented with different doses of curcumin, i.e. from 50 to 300 mg cur/kg BW (Fig. 1b). The brain water content was reduced significantly in all the curcumin doses tested as compared to control (hypoxia-exposed rats without curcumin). Among the doses tested in the present study, the maximum or significant reduction in transvascular leakage or edema index was obtained at 100 mg cur/kg BW, therefore, this dose was considered as the optimum dose. Hence, further experiments were carried out using 100 mg cur/kg BW as the optimum dose. Rats supplemented with 100 mg cur/kg BW under hypoxia showed a significant reduction (*p* < 0.001) in the brain water content (3.53 ± 0.58 W/D ratio) as compared to hypoxia-exposed rats without curcumin (7.1 ± 1.0 W/D ratio). However, the same dose under normoxia did not alter the brain water content compared to control (normoxia) (data not shown).

### Haematological analysis

Table 1 shows the changes in haematological parameters of the different groups of animals. A significant increase (*P* < 0.001) in WBC, RBC, lymphocyte, monocytes, granulocytes, MCV and Hb were observed in the 24-h hypoxia-exposed rats as compared to normoxia. Curcumin at 100 mg/kg BW administration to rats 1 h prior to 24-h hypoxia resulted into a significant reduction (*p* < 0.001) in the WBC and lymphocyte as compared to 24-h hypoxia without curcumin. However, the levels of monocyte, granulocyte, RBC, MCV and Hb were maintained at reduced levels (*p* < 0.001) under curcumin treatment compared to hypoxia-exposed animals. Further, normoxic animals treated with curcumin showed these values more or less similar to that of control.

**Table 1:** Haematological analysis

Parameters	Normoxia	Hypoxia	Normoxia + curcumin	Hypoxia + curcumin
WBC (million/mm^3^)	7.1 ± 1.8	14.6 ± 2.1*	8.3 ± 1.2	7.2 ± 1.9^#^
Lymphocyte (%)	87 ± 3.4	99.7 ± 3.1*	88.7 ± 3.2	87.1 ± 1.7^#^
Monocyte (%)	3.9 ± 0.5	8.9 ± 1.1*	3.5 ± 0.8	3.9 ± 1.8^#^
Granulocyte (%)	3.39 ± 0.6	12 ± 2.1*	3 ± 0.4	4.1 ± 1.7^#^
RBC (million/mm^3^)	7.5 ± 1.4	15.8 ± 1.2*	7.9 ± 0.5	7.98 ± 2.2^#^
MCV (%)	66.6 ± 3.5	76.9 ± 3.5*	64 ± 2.9	65.8 ± 2.9^#^
Hb (g/dl)	13.2 ± 1.9	16.8 ± 1.9*	13 ± 1.2	14.4 ± 2.5^#^

Lymphocytes, monocytes and granulocytes expressed in %

*WBC* white blood cells (million/mm^3^), *RBC* red blood cells (million/mm^3^), *MCV* mean cell volume (% volume), *Hb* Haemoglobin (g/dl)

*p < 0.01 compared to normoxia; ^#^ *p* < 0.001 compared to hypoxia

### Brain nuclear factor kappa B (NF-κB) p65 protein expression and the proteins involved in its activation

**Fig. 2:**
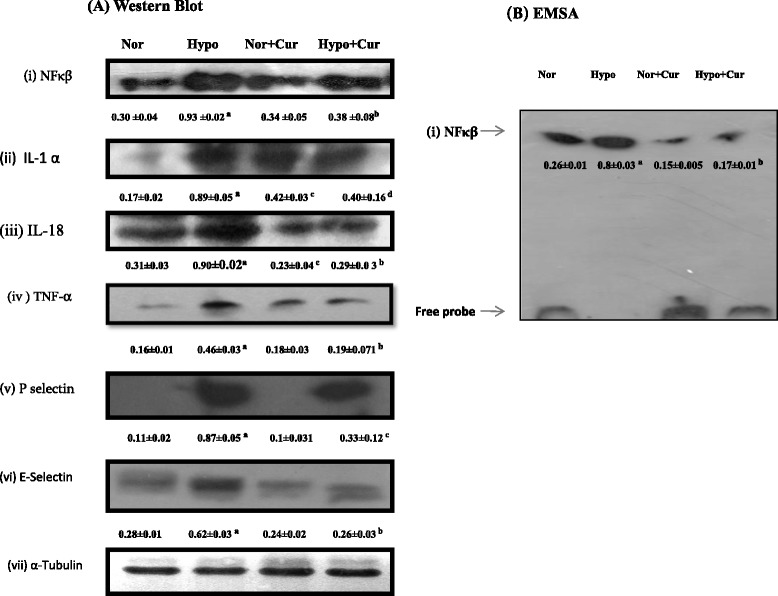
Protein expression pattern of NF-κB and related genes in the brain homogenate of rats exposed to hypobaric hypoxia for 24 h at 7620 m. **a** Representing Western blot analysis of (i) NF-κB, (ii) IL-1, (iii) IL-18, (iv) TNF-α, (v) P-selectin and (vi) E-selectin proteins in the brain of rats under hypoxia. **b** Depicts the NF-κB-DNA-binding activity. Nuclear extracts were prepared and used to analyse the NF-κB-DNA binding by EMSA. The *arrow* indicates the position of NF-κB and free probe. The enhanced NF-κB and its downstream genes were downregulated by curcumin treatment prior to hypoxia exposure. The densitometry analyses were represented below their respective analyses. Values are mean ± SD (*n* = 6). ^a^ *p* < 0.001 compared with normoxia (0 h) group; ^b^ *p* < 0.001 compared with hypoxia (24 h) group; ^c^ *p* < 0.05 compared with normoxia (0 h) group; ^d^ *p* < 0.05 compared with hypoxia (24 h) group. *Nor* normoxia, *Hypo* hypoxia, *Cur* curcumin (100 mg/kg BW)

Curcumin modulates the hypoxic expression of redox sensitive transcriptional factor-NF-κB:

Hypoxic exposure resulted into a significant upregulation of NF-κB (nearly threefold ↑) expression (Fig. 2a, i) compared to control; whereas rats supplemented with curcumin under hypoxia showed reduction in expression of NF-κB (2.44-fold ↓, *p* < 0.001) in nuclear extract isolated from the brain of rats compared to hypoxia-exposed rats.

### Brain NF-κB-DNA binding assay

To further confirm whether increased translocation of NF-κB also results in to increased DNA binding activity, electro mobility shift assays (EMSA) were performed using highly specific biotinylated-oligonucleotide probe. The results revealed that nearly threefold increase in NF-κB DNA (*p* < 0.001) binding activity in the brain of rats exposed to hypoxia over control animals. The rats receiving curcumin showed reduced (↓4.7-fold) DNA binding activity under hypoxia compared to control (hypoxia-exposed rats without curcumin supplementation) (Fig. 2b (i)).

### Expression of some genes reflected to be involved in inflammation (IL-1α, IL-18, TNF-α, E-selectin and P-selectin)

Hypoxic exposure resulted into enhanced levels of cytokines like IL-1α, IL-18 and TNF-α (fold increase 5.2 ↑, 2.9↑ and 2.8↑, respectively). Further, we observed that, hypoxic exposure resulted into upregulation of selectins like P-selectin and E-selectin (fold increase 8↑ and 2.2↑, respectively) in the brain of rats compared to control. However, we noticed that IL-1 α (2.2 ↓), IL-18 (3.1↓) and TNF-α (2.4↓) expressions were reduced (fold reduction) appreciably in response to 100 mg Cur/kg BW treatment under hypoxia as compared to control (Fig. 2a (ii, iii and iv), respectively). Similarly, the expressions of cell adhesion molecules like P-selectin (2.63↓) and E-selectin (2.38↓) were reduced appreciably in response to supplementation of curcumin in the brain of rats exposed to hypoxia (Fig. 2a (v and vi)) compared to control (24-h hypoxia).

#### ELISA

**Fig. 3:**
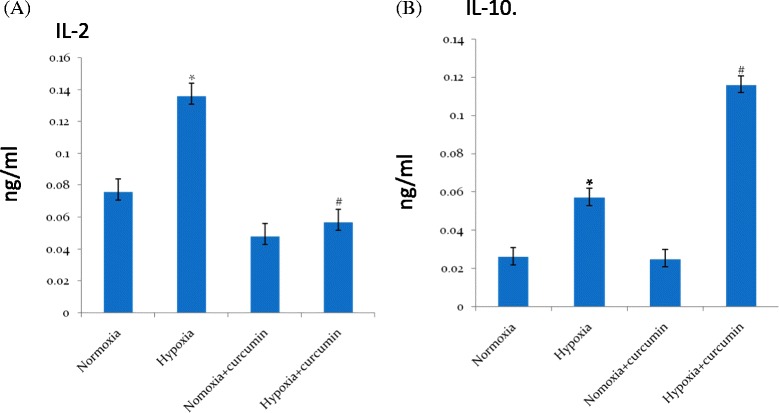
ELISA of (**a**) pro-inflammatory molecule IL-2 and (**b**) anti-inflammatory molecule IL-10 in the brain homogenates of rats exposed to hypoxia at 7620 m for 24 h. Hypoxic exposure resulted in to a significant increase in IL-2 followed by significant decrease in IL-10 levels in the brain of rats compared to control. However, curcumin treatment reversed the condition from pro-inflammatory to anti-inflammation by reducing IL-2 levels followed by enhanced IL-10 levels in the brain of rats under hypoxia compared to control (hypoxia) 24 h. Values are mean ± SD (*n* = 6). ^*^ *p* < 0.001 compared with normoxia (0 h) group. ^#^ *p* < 0.05 compared with hypoxia (24 h) group. *Nor* normoxia, *Hypo* hypoxia, *Cur* curcumin (100 mg/kg BW)

Exposure to hypoxia showed an increase of plasma IL-2 levels (0.131 ± 0.08 ηg/ml, Fig. 3a) followed by a significant fall in IL-10 levels (0.056 ± 0.06 ηg/ml, Fig. 3b) in rats compared to control (0.073 ± 0.03 ηg/ml and 0.025 ± 0.02 ηg/ml respectively). Whereas, preconditioning of rats with curcumin showed enhanced levels of IL-10 (0.196 ± 0.04 ηg/ml) followed by appreciable fall in IL-2 levels (0.057 ± 0.06 ηg/ml) compared to control (24-h hypoxia, *p* < 0.001). However the concentrations of IL-10 levels were not modified up on curcumin administration in normoxic group (Normoxia + curcumin) compared to control (Normoxia) animals (Fig. 3b). Together these cytokine data indicates, convincing evidence that, the curcumin supplementation prior to hypoxic exposure significantly reduced the pro-inflammatory cytokine followed by enhanced anti-inflammatory cytokine milieu under hypoxia.

### Brain HIF-1α, EMSA of HIF-1α and one of its regulated genes (VEGF)

#### Anti-inflammatory effect of curcumin is associated with HIF-1α stabilisation

**Fig. 4:**
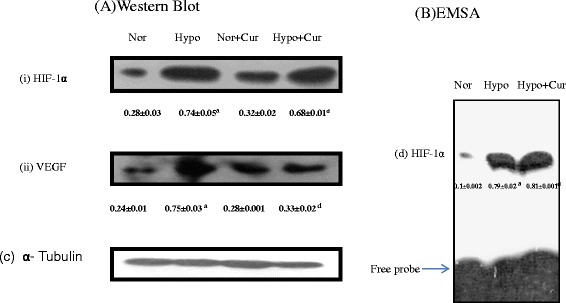
Protein expression pattern of HIF-1α and related genes in the brain homogenate of rats exposed to hypobaric hypoxia for 24 h at 7620 m. **a** Represents Western blot analysis of (i) HIF-1α and (ii) VEGF proteins in the brain of rats. **b** Depicts the HIF-1α-DNA-binding activity. Nuclear extracts were prepared and used to analyse the HIF-1-DNA binding by EMSA. The *arrow* indicates the position of HIF-1α and free probe. Hypobaric hypoxia increased the HIF-1α and VEGF protein levels in the brain of rats. Whereas pre-treatment of rats with curcumin and exposed to hypoxia maintained both HIF-1α and VEGF values compared to that of control. Values are mean ± SD (*n* = 6). ^a^ *p* < 0.001 compared with normoxia (0 h) group; ^d^ *p* < 0.05 compared with hypoxia (24 h) group. *Nor* normoxia, *Hypo* hypoxia, *Cur* curcumin (100 mg/kg BW)

HIF-1α plays a critical role in oxygen homeostasis. As expected, exposure to hypoxia significantly upregulated the HIF-1α (2.6-fold ↑) followed by enhanced upregulation of VEGF (3.1-fold ↑) in the brain homogenates of rats compared to control rats. Surprisingly, we have found that curcumin stabilised the HIF-1α expression (Fig. 4a (i)) levels (1.08-fold reduction) in the brain of rats under hypoxia. Thus, we examined the possibility that curcumin can alter the protein-DNA binding activity of HIF-1α in the brain homogenate of rats compared to control (24-h hypoxia) (Fig. 4b (d)). Further, we have determined the expression of one of the genes regulated by HIF-1α, i.e. VEGF in the brain homogenate of rats supplemented with curcumin under hypoxia. VEGF (Fig. 4a (ii)) expression was found to be maintained (2.27-fold decrease) indicating the role of curcumin in sustaining the VEGF expression in the brain of rats under hypoxia. Animals receiving curcumin under normoxia did not show any significant change in HIF-1α and VEGF levels in the brain tissue (Fig. 4a) indicating that curcumin (at this concentration) in normoxic conditions does not alter the body’s oxygen homeostasis mechanism.

#### Proteins involved in fluid clearance

**Fig. 5:**
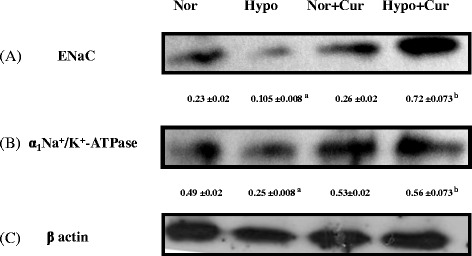
Effect of curcumin (100 mg/kg BW) on the ion channel expression in the brain homogenate of rats exposed to hypobaric hypoxia at 7620 m for 24 h. **a** Represents Western blot analysis of ENaC protein, **b** Na^+^/K^+^-ATPase protein expression and **c** β-actin protein expression. Hypoxia downregulated the ENaC and Na^+^/K^+^-ATPase which were appreciably enhanced by curcumin administration 1 h prior to hypoxia exposure. ^a^ *p* < 0.001 compared with normoxia (0 h) group; ^b^ *p* < 0.001 compared with hypoxia (24 h) group; *Nor* normoxia, *Hypo* hypoxia, *Cur* curcumin (100 mg/kg BW)

Exposure to hypoxia showed a fall in the brain Na^+^/K^+^-ATPase (2.2-fold decrease) expression over normoxia. The levels of Na^+^/K^+^-ATPase expression were found to be upregulated (Fig. 5b) (6.8-fold increase, *p* < 0.05) on curcumin administration. Similarly, curcumin appreciably maintained the higher ENaC protein expression (2.24-fold ↑, *p* < 0.001) in the brain of rats under hypoxia (Fig. 5a). Curcumin administration under normoxia did not alter the levels of both Na^+^/K^+^-ATPase and ENaC protein expressions compared to control (normoxia).

To ensure that equal concentration of protein had been loaded, β-actin and α-tubulin protein expressions were determined in the brain homogenate by Western blotting.

#### Tight junction proteins

**Fig. 6:**
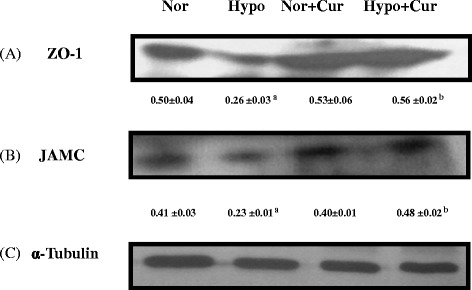
Effect of curcumin (100 mg/kg BW) on the tight junction proteins expression in the brain homogenate of rats exposed to hypobaric hypoxia at 7620 m for 24 h. Western blot analysis of (**a**) ZO-1, **b** JAMC and (**c**) α-tubulin. Rats exposed to hypoxia showed downregulation of brain JAMC and ZO-1 protein expression levels compared to control. Curcumin restored the altered tight junction proteins integrity under hypoxia. ^a^ *p* < 0.001 compared with normoxia (0 h) group; ^b^ *p* < 0.001 compared with hypoxia (24 h) group. *Nor* normoxia, *Hypo* hypoxia, *Cur* curcumin (100 mg/kg BW)

Endothelial cells of cerebral microvasculature serve as a frontline defence, protecting neurons and glial cells from harmful insult. Therefore, we further evaluated the functional and molecular changes in the brain micro vessel endothelial cells and correlated loss in tight junction proteins integrity with transvascular leakage studies. Hypoxia induced nearly 46 % increase in transvascular leakage indicating disruption at TJ protein complexes. This effect was significantly reduced (nearly 51 %) with curcumin treatment. Hypoxic exposure resulted in diminished levels (nearly 2.0-fold ↓) of the brain ZO-1 protein expression. The ZO-1 expression levels were found to be appreciably upregulated in curcumin-treated rats under hypoxia (2.15-fold ↑) (24-h hypoxia) (Fig. 6a). However, JAMC levels were upregulated (2.08-fold ↑) under curcumin treatment in the brain of rats during hypoxic exposure (Fig. 6b).

#### Immunohistochemical studies of tight junction proteins

**Fig. 7:**
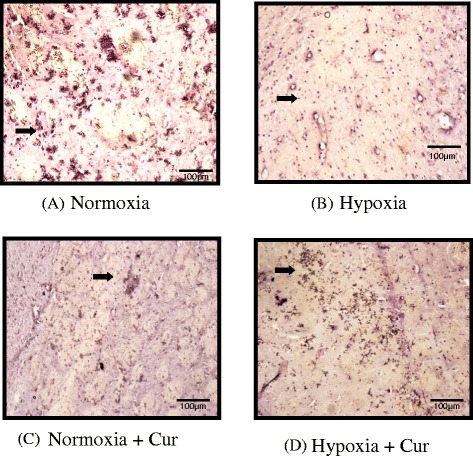
Immunohistochemical analysis showing the effect of curcumin (100 mg/kg BW) on the expression of claudin 4 in the brain of rats exposed to hypobaric hypoxia at 7620 m, for 24 h (**a–d**). Exposure to hypobaric hypoxia showed reduced expression of claudin 4 levels under hypoxia. Whereas, preconditioning with curcumin significantly maintained higher claudin 4 levels under hypoxia. Surprisingly, curcumin did not alter the claudin 4 levels under normoxia compared to control. The figure is representative of at least 4–5 animals from each group (40×). *Nor* normoxia, *Hypo* hypoxia, *Cur* curcumin (100 mg/kg BW)

**Fig. 8:**
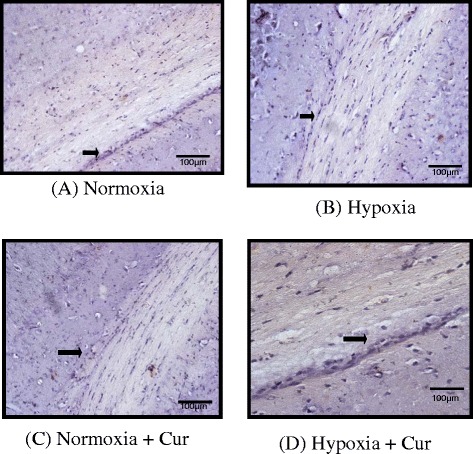
Immunohistochemical analysis showing the effect of curcumin (100 mg/kg BW) on the expression of claudin 5 in the brain of rats exposed to hypobaric hypoxia at 7620 m for 24 h (**a–d**). Hypoxia showed poor expression of claudin 5 in the brain of rats under hypoxia. However, it was noticed that curcumin administration resulted in to a significant upregulation of claudin 5 under hypoxia. Curcumin did not modify the claudin 5 levels under normoxia. The figure is representative of at least 4–5 animals from each group (40×). *Nor* normoxia, *Hypo* hypoxia, *Cur* curcumin (100 mg/kg BW)

It was observed that claudin 4 and claudin 5 levels were significantly downregulated under hypoxia. Preconditioning with curcumin significantly maintained higher levels of claudins 4 and 5 under hypoxia. Surprisingly, curcumin did not alter the claudins 4 and 5 levels under normoxia compared to control (Figs. 7 and 8, respectively).

### Changes in the brain histopathology

**Fig. 9:**
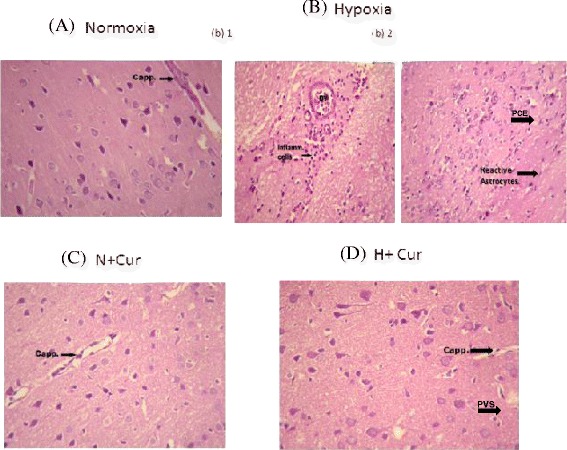
Histological examination of the different groups of rat brain tissues (haematoxylin-eosin staining at 40×). **a** Photomicrograph from section of the cerebral cortex of the animal under normoxic condition showing normal arrangement and structure of cortical neurons. A cerebral capillary with normal configuration is seen at the *upper right* corner. **b** Photomicrograph from section of the cerebral cortex of the animal exposed to 24 h of hypoxia showing numerous inflammatory cells around a blood vessel (*BV*). Photomicrograph from another area of section of cerebral cortex of animal exposed to 24 h of hypoxic condition showing scattered inflammatory cells (*b1*) and reactive astrocytes (*b2*). **c** Normoxic animals treated with curcumin showed normal configuration in the brain tissue sections. **d** Photomicrograph from section of the cerebral cortex of the animal exposed to 24 h of hypoxic condition, treated with 100 mg/kg BW of curcumin, showing normal arrangement and structure of cortical neurons without inflammatory cells. A cerebral capillary with normal configuration is seen in the *right half* (*d*). *Cap* capillary structure, *BV* blood vessels, *Inflam cells* inflammatory cells, *PVS* perivascular space, *PCE* pericellular edema, *N* normoxia, *H* hypoxia, *Cur* curcumin

Histological examination of the brain tissues of different groups of animals is depicted in Fig. 9. Brain tissue sections of (Fig. 9a) normoxic animals showed a normal arrangement and structure of cerebral capillaries with normal configuration. However, sections of the animals exposed to 24-h hypoxia showed numerous inflammatory cells around a blood vessel of the cortical neurons indicating vascular leakage (Fig. 9b (1)), while another section of the same group of animals showed scattered inflammatory cells, reactive astrocytes and pronounced widening of pericellular spaces around the cortical neurons indicating pericellular edema (PCE) manifesting the clear involvement of inflammation as well as edema in the 24-h hypoxia-exposed brain samples (Fig. 9b (2)). When the effect of curcumin was seen in the normoxic animals, the brain tissue sections showed normal configuration (Fig. 9c). Brain tissue sections of the curcumin pretreated hypoxia-exposed animals resulted in a very mild widening of the perivascular space (PVS) and no widening of the pericellular space, showing an almost negligible edema of the cerebellum (Fig. 9d).

## Discussion

Traditionally, turmeric and other curcuminoids have been used in therapeutic preparations for various ailments in different parts of the world. Numerous therapeutic effects of curcumin/turmeric have been confined to modern scientific research [19–23]. Hypoxia is associated with inflammatory diseases, such as acute lung injury or cardio respiratory conditions with low cardiac output. However, the mechanisms directly linking hypoxia to inflammation remain unclear. Therefore, we undertook an investigation to identify the molecular targets causing vascular leakage through impaired tight junction proteins integrity and failure in fluid reabsorption in the brain of rats; and surprisingly, these changes were mitigated by administering curcumin as a prophylactic drug. The results of the present study indicate that curcumin modulates the oxygen-dependent transcription of NF-κB and HIF-1α; the two genes involved in regulation of adaptive responses (Na^+^/K^+^-ATPase and ENaC) and also decrease in the brain inflammation leading to reduction in cerebral edema by retaining the tight junction proteins integrity in the brain of rats.

### Role of curcumin in modulating the haematological changes under high-altitude hypoxia

**Fig. 10:**
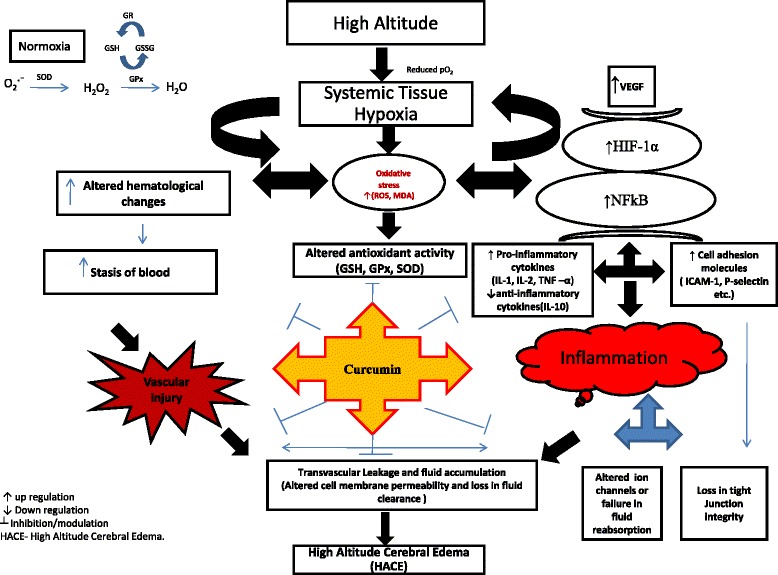
Schematic representation of possible mechanism of action of curcumin against HACE as prophylactic drug. Exposure to hypobaric hypoxia increased the transvascular leakage leading to fluid build-up in the brain due to upregulated NF-κB and HIF-1α and their downstream genes that play a major role in causing inflammation (pro-inflammatory cytokines and cell adhesion molecules) and vascular leakage (VEGF). Further, hypobaric hypoxia exposure impaired the Na^+^/K^+^-ATPase and ENaC expression followed by altered tight junction protein (ZO-1, JAMC, claudin 4 and claudin 5) integrity in the brain of rats; whereas prophylactic treatment of rats with curcumin reversed the changes brought by hypobaric hypoxic treatment. *Inverted letter T* inhibition, *upwards arrow* upregulation, *downwards arrow* downregulation, *HACE* high-altitude cerebral edema

On exposure to high altitude, a number of physiological responses occur with changes in ventilatory responses as well as haematological parameters such as haemoglobin concentration (Hb), red blood cell (RBC) and packed cell volume (PCV) which contribute to increase the oxygen-carrying capacity of the blood as a compensatory response to high-altitude hypoxia [35]. There are reports inferring that immediately after arrival to altitude, physiological adaptations occur within 4 h of hypoxic exposures, which will last for many weeks if exposure continues [36]. It has also been reported that on the first day of arrival at altitude, plasma volume decreases progressively and an increase in erythrocyte production is observed at altitude due to an augmentation of erythropoietin level [37–39]. In the present study, when the rats were exposed to an altitude of 7620 m for 24-h durations, the haematological parameters viz., WBC, lymphocytes, RBC and Hb, showed a significant rise. However, administration of curcumin prior to hypoxia exposure was found to modulate the haematological parameters with respect to a decline in the level of WBC and lymphocyte along with statistically significant changes in RBC and Hb level as compared to hypoxia-exposed rats without curcumin. Modulatory changes that occurred up on curcumin administration to the rats under hypoxia in the present study might be due to the functional consequences of physiological responses to hypoxia exposure, which might further lead to improved oxygen-carrying capacity, if not fully, but at least to compensate for the decrease in available oxygen in order to maintain the cellular homeostasis at molecular level. These changes were sequentially depicted (Fig. 10) and discussed in the ensuing paragraphs.

### Curcumin facilitates the cellular and systemic responses to hypoxic stress via cross talk between NF-κB and HIF-1α

Several studies have revealed that hypoxia inducible factor (HIF-1α) plays an important role in the evolution and propagation of the inflammatory process [40–42]. In order to check this property, in our present study, we have determined the HIF-1α level in the brain homogenate of rats under hypoxia. The results showed that not only the activation of cerebral NF-κB was inhibited by curcumin treatment but also showed stabilised HIF-1α protein expression. Furthermore, we also observed that HIF-1α regulated gene VEGF, which was increased under hypoxia, was significantly downregulated, rather maintained more or less similar to that of control in curcumin-treated rats. Curcumin is an in vivo inhibitor of VEGF upregulation, thereby controlling the angiogenesis [43]. VEGF expression can be regulated through dual independent mechanisms involving HIF-1α directly (via HIF-1α-VEGF promoter) and also through NF-κB activation [44]. It seems that evolutionary conserved HIF-1α is regulated by NF-κB [40]. It was reported by Jordi et al. [45] that Ikappa kinase (IKK) β in different cell types demonstrate that NF-κB is a critical activator of HIF-1α in macrophages, responding to bacterial infection in the brain and liver of hypoxic animals. IKKβ deficiency results in defective induction of various HIF-1α targets genes including VEGF in mice. Hence, IKKβ provides an important physiological link between the hypoxic response to innate immunity and inflammation, two ancient stress response systems. Moreover, it was reported by Xiong et al. [46] that treatment with bacterial lipopolysaccharide (LPS) or with CD40 L stimulates VEGF production in human primary macrophages. This effect has been reported to be NF-κB dependent, because it is sensitive to the overexpression of the inhibitory protein Ikappa B (IKB) α [47]. Whereas, Robert et al. [48] have revealed an interesting observation that VEGF expression in macrophages is regulated by Liver X factor (LXR) which is independent of HIF-1 activation and also did not require the previously characterised hypoxia response element in the VEGF promoter. The VEGF is primarily known as the inducer of angiogenesis, but this cytokine has roles in vascular permeability and haematopoietic cell development and differentiation [49]. VEGF is important in both inflammation and repair and is critical in resolution of primary process of wound healing. It is well known that higher expression of HIF-1α and its down regulatory gene expression of VEGF are necessary in maintaining the oxygen homeostasis in the cells during hypoxia, but excess is detrimental, as increased VEGF levels alter permeability markedly exacerbating the high permeability cerebral edema [6]. Our study suggests that activation of NF-κB and HIF-1α in this context may also lead to increased production of VEGF. However, it was reported that inhibition of NF-κB correlates with downregulation of VEGF mRNA [50] which may be mediated through HIF-1α. On the other hand, the enhanced expression of NF-κB levels seems to be positively correlated with increased paracellular permeability as these changes were associated with alterations in tight junction proteins. This indicates that inflammation plays a significant role in tight junction protein disruption under hypoxia. Recently, we demonstrated that NF-κB and oxidative stress contribute in transvascular leakage leading to fluid influx in the brain [15]. Curcumin significantly downregulated the higher VEGF levels under hypoxia. Our results are in agreement with the results reported earlier [43]. This perhaps helps in maintaining oxygen homeostasis by facilitating acclimatisation thereby abridging the fluid influx into the brain of rats exposed to hypoxia under current experimental conditions. A study by Witt et al. [51] suggested that transcription factors such as HIF-1α and NF-κB are upstream mediators of TJ protein alterations during hypoxia and hypoxia re-oxygenation which may involve VEGF induction and expression. In this regard at high altitude, lack of HIF-1α response or excessive expression of HIF-1α results in the lack of ability to adapt to hypoxia. Therefore, these results clearly reveal the importance of HIF-1α stabilisation and downregulation of NF-κB, with enhanced anti-inflammatory molecule (IL-10) through curcumin preconditioning, leading to reduction in transvascular leakage in the brain of rats.

There is a major concern that the low serum concentrations of curcumin normally observed in rodents and humans may not reach a particular organ in sufficient concentrations to have an effect. Recent studies, however, have suggested a favourable tissue distribution of curcumin. At least two studies suggest that curcumin is a fluorescent compound that binds to amyloid deposits. Garcia-Alloza et al. [52] were able to use multiphoton microscopy to demonstrate that curcumin administered systematically in mice crossed the blood brain barrier, bound to amyloid plaque in the brain and reversed the existing amyloid pathology using fluoropropyl substituted synthetic curcumin. Ryu et al. [53] showed that curcumin was taken up by the brain. Importantly, curcumin does not block the pathway totally, but only, downregulate the overactive pathway to basal levels. In vitro and in vivo and human clinical studies have all established curcumin’s promise and revealed its therapeutic value. Cheng et al. [54] reported that no treatment-related toxicity was observed up to 8 g daily in phase I clinical trials, but beyond this dose, the bulk volume of the drug was unacceptable to the patients. Similarly, several other clinical trials have reported use of curcumin on various disease conditions viz, cancer [55], familial adenomatous polyposis [56], tropical pancreatic cancer [57], inflammatory bowel disease [58, 59], gall bladder function [60], psoriasis [61] and helicobacter pylori infection [62].

### Adaptive mechanisms under hypoxia were well maintained by curcumin administration

Decreases in Na^+^/K^+^-ATPase activity have been reported during cerebral ischemia leading to cellular edema and ultimately contributing to cell death [63]. This is probably due to the fact that ischemia or hypoxia induces energy crisis [64], increases reactive oxygen species (ROS) generation [65] along with release of endogenous inhibitors of Na^+^/K^+^-ATPase [66]. So, it seems that reduced Na^+^/K^+^-ATPase activity is a common event in a number of neuronal degenerative or metabolic diseases. Earlier reports reveal that increased ROS results into increased NF-κB activity in the brain of rats [15]. In the present study, we found that the increase in NF-κB levels followed by increased proinflammatory cytokines (IL-1, IL-18 and TNF- α) cell adhesion molecules (P-selectin and E-selectin) and significant reduction in IL − 10 levels resulted in to reduction in the brain Na^+^/K^+^-ATPase and ENaC levels. Heerlein et al. [67] reported that making use of O_2_ for protein synthesis during hypoxic environment falls up to 30 % when hypoxia is extended to 24 h which is not reversible. Perhaps this could be the reason in the present study that the brain Na^+^/K^+^-ATPase levels were significantly reduced during 24 h of hypoxia exposure, whereas curcumin restored their levels. Further, curcumin appreciably maintained ENaC levels under hypoxia. These results indicate that curcumin prophylaxis is able to facilitate the adaptive mechanisms by enhancing the expression of ENaC proteins and Na^+^/K^+^-ATPase probably through attenuation of NF-κB and stabilisation of HIF-1 α thereby controlling the fluid influx leading to enhanced fluid reabsorption mechanism in the brain.

### Tight junction integrity in the brain of rats was retained by curcumin prophylaxis

In this study, we examined the hypoxia effect and pre-treatment of rats with curcumin and its effects on paracellular permeability and TJ integrity. One of the important TJ proteins is ZO-1, which was found to be decreased significantly (*p* < 0.001) under hypoxia. Fischer et al. [68] revealed that hypoxia induces the alterations in ZO-1 proteins. Similar results were observed in the present study. However, we noticed that hypoxia induced decrease in TJ integrity was recovered by curcumin administration. This recovery with curcumin correlates with changes in protein expression of other TJ proteins. Increased TJ protein synthesis might have enhanced the cell-to-cell response in cyto-architectural alternations and therefore led to stop paracellular permeability. The low molecular weight and polar structure of curcumin allows it to penetrate the blood–brain barrier effectively. Our study was supported by previous studies, where Mark and Davis [9] reported that hypoxic insult to the brain endothelial cells induces restructuring of TJ and cyto-architecture (i.e. changes in protein localisation) that correlates with observed functional changes, i.e. increased paracellular permeability. These authors further observed that exposure to hypoxic environment may trigger re-location of some TJ proteins from the plasma membrane to the cytoplasm. These results preclude that cerebral micro vessel endothelial cells undergo several molecular and functional changes during hypoxia exposure. It is true that it is not the only low levels of O_2_ (hypoxia), but several mediators including pro-inflammatory cytokines (IL-1 α, TNF-α) and VEGF contribute to the disruption of BBB [41, 69–71]. The increase in IL-1 α, IL-2, IL-18, TNF-α, P-selectin and E-selectin in the present study was self explanatory to hypothesise the contribution of pro-inflammatory cytokine milieu in the development of HACE and curcumin preconditioning abolished these changes. This might be true that curcumin physically binds to a wide range of cellular proteins including structural proteins and metabolic enzymes [72]. We observed that, finally, it is the inflammation and not only the oxidative stress that contributes in causing fluid influx by disturbing the tight junction integrity in the brain. Immunohistochemical studies showed that the hypoxia-decreased claudin 4 and claudin 5 proteins were upregulated by curcumin prophylaxis. Histopathological studies have further strengthened our results, showing reduction in edema which correlates with the observed changes in BBB permeability. Indeed, we observed that the increased VEGF levels were positively correlated with alterations in ZO-1, JAMC and claudin 4 and claudin 5 proteins. Therefore, the results of this study clearly indicate that hypoxia-induced changes in paracellular permeability may be due to neuroinflammation and that curcumin reverses these effects.

Hypoxic cerebral vasodilation appears to be a necessary ingredient, but does not per se explain the development of brain edema. However, it was reported earlier that despite normal cerebral oxygenation and global cerebral metabolism, vasogenic edema develops in humans who became moderately ill with AMS/HACE during 24 h or more of hypoxic exposure [73]. Although dexamethasone (a recommended drug against HACE) in high doses can completely remove the symptoms of AMS and cerebral edema, but it does not help in acclimatisation, and moreover, the drug has many side effects of steroid therapy [74, 75] which may indicate that dexamethasone cannot be used for longer durations against HACE. Additionally, AMS symptoms seem to persist when the drug is stopped. Therefore, it seems reasonable to hypothesise that curcumin is able to inhibit the neuronal inflammation (↓NF-κB) and maintained oxygen metabolism (stabilising HIF-1α) by facilitating acclimatisation along with enhanced tight junction proteins integrity and ion channels expression thereby controlling the fluid influx in to the brain under hypoxia. In complex multifactorial illness, i.e. HACE, an agent that can act at a number of different cellular levels offers perhaps a better chance of effective prophylaxis or treatment. This suggests that preconditioning with curcumin might be more effective in rat model of HACE and also may provide similar protection in prevention of HACE in humans. Moreover, the safety, low cost and already proven efficacy of this age-old natural medicine makes it a promising agent for the prevention of high-altitude cerebral edema.

## Conclusions

In conclusion, we report that hypoxia induces increased paracellular permeability leading to fluid accumulation in the brain. This profound effect was significantly reduced by prior curcumin supplementation and these functional changes might be associated with downregulation of NF-κB (inflammation) and lowering the over expression of HIF-1α (oxygen homeostasis) thereby maintaining TJ proteins integrity followed by increased ion channels expression. This was the first report examining the prophylactic administration of curcumin on protein expression of active ion channels and TJ proteins integrity in the cerebral vasculature under hypobaric hypoxia conditions. Understanding the cellular mechanisms induced by hypoxia and prophylactic treatment with curcumin that alter BBB permeability will certainly contribute to develop pharmacotherapeutics for treatment or prevention of decreased O_2_ conditions as seen at high altitudes or in some diseased conditions.

## Abbreviations

ANOVA: analysis of variance
BW: body weight
BV: blood vessel
Cap: capillary structure
Cur: curcumin
DMSO: dimethyl sulphoxide
DNA: deoxyribonucleic acid
EDTA: ethylene diamine tetra acetic acid
ELISA: enzyme-linked immunosorbent assay
ENaC: endothelial sodium channel
H: hypoxia
HACE: high-altitude cerebral edema
HIF-1α: hypoxia-inducible factor 1 alpha
Hg: mercury
HRP: horse radish peroxidases
ICAM: intercellular adhesion molecule
IKB: Ikappa B
IKK: ikappa kinase
IL: interleukin
JAMC: junctional adhesion molecule C
LPS: lipopolysaccharide
LXR: liver X factor
MW: molecular weight
N: normoxia
Na^+^/K^+^-ATPase: sodium potassium-adenosine triphosphatase
NSAID: non steroidal anti-inflammatory drugs
NF-κB: nuclear factor-kappa B
PBS: phosphate buffered saline
PCE: pericellular edema
PVS: perivascular space
r.f.u.: relative fluorescence unit
ROS: reactive oxygen species
RT: room temperature
TNF-α: tumour necrosis factor
UFAW: Universities of Federation for Animal Welfare
VEGF: vascular endothelial growth factor
ZO-1: Zonula occludin 1
